# Biological Response Profiling Reveals the Functional Differences of Main Alkaloids in Rhizoma Coptidis

**DOI:** 10.3390/molecules26237389

**Published:** 2021-12-06

**Authors:** Lan Xie, Shanshan Feng, Xiaoling Zhang, Wenlong Zhao, Juan Feng, Chengmei Ma, Ruijun Wang, Weifang Song, Jing Cheng

**Affiliations:** 1 Medical Systems Biology Research Center, School of Medicine, Tsinghua University, Beijing 100084, China; xielan@tsinghua.edu.cn (L.X.); fengjuan@tsinghua.edu.cn (J.F.); 2 National Engineering Research Center for Beijing Biochip Technology, Beijing 102206, China; fengshanshanyl@hec.cn (S.F.); xiaolingzhang@capitalbio.com (X.Z.); wenlongzhao@capitalbio.com (W.Z.); chengmeima@capitalbio.com (C.M.); 3 Department of Pathophysiology, Fenyang College, Shanxi Medical University, Fenyang 032200, China; ruijunwang@capitalbio.com (R.W.); bsswf@163.com (W.S.)

**Keywords:** rhizoma coptidis, alkaloid, berberine, coptisine, transcriptome analysis

## Abstract

Rhizoma Coptidis (RC) is a widely used traditional Chinese medicine. Although modern research has found that some alkaloids from RC are the pharmacologically active constituents, the differences in their biological effects are not completely clear. This study analyzed the differences in the typical alkaloids in RC at a systematic level and provided comprehensive information on the pharmaceutical mechanisms of the different alkaloids. The ethanol RC extract (RCE) was characterized using HPLC assay. HepG2, 3T3-L1, and RAW264.7 cells were used to detect the cytotoxicity of alkaloids. Transcriptome analyses were performed to elucidate the cellular pathways affected by RCE and alkaloids. HPLC analysis revealed that the typical alkaloids of RCE were berberine, coptisine, and palmatine. Coptisine and berberine displayed a stronger inhibitory effect on cell proliferation than palmatine. The overlapping ratios of differentially expressed genes between RCE and berberine, coptisine, and palmatine were 70.8%, 52.6%, and 42.1%, respectively. Pathway clustering analysis indicated that berberine and coptisine possessed a certain similarity to RCE, and both compounds affected the cell cycle pathway; moreover, some pathways were uniquely enriched by berberine or coptisine. Berberine and coptisine had different regulatory effects on genes involved in lipid metabolism. These results provide comprehensive information on the pharmaceutical mechanisms of the different RC alkaloids and insights into their better combinatory use for the treatment of diseases.

## 1. Introduction

Rhizoma Coptidis (RC, huanglian in Chinese) is a widely used traditional Chinese medicine that refers to the dried roots of *Coptis chinensis* Franch, *Coptis deltoidea* C. Y. Cheng et Hsiao, and *Coptis teeta* Wall, according to the Chinese Pharmacopoeia [1]. RC has properties of clearing heat, drying dampness, and detoxification and has been used for centuries in the treatment of gastrointestinal infections, inflammation, and liver diseases in Chinese medical practice [2].

Chemical investigations have revealed multiple secondary metabolites in RC, among which alkaloids are the major components [2]. The main alkaloids in RC include berberine, coptisine, palmatine, epiberberine, jatrorrhizine, columamine, magnoflorine, and worenine [3,4]. In the Chinese Pharmacopoeia, berberine, coptisine, palmatine, and epiberberine are designated as quality markers for RC.

Modern research has demonstrated that RC and its bioactive alkaloids have various pharmacological activities. One of the most important pharmacological activities of RC is its antimicrobial activity against bacteria, viruses, fungi, protozoans, helminths, and chlamydia [5]. In addition, RC has been shown to have anti-tumor activity, and this activity has been validated in various tumor types, including liver cancer, breast cancer, melanoma, colon cancer, and lung cancer [6]. Recently, RC has been reported to have anti-inflammatory, antioxidant, anti-diabetic, lipid-lowering, and neuroprotective effects [2,7,8]. Among the alkaloids in RC, berberine is the most abundant [3] and has been studied intensively. Berberine can replicate the anti-tumor and anti-inflammatory effects of RC [9]. Additionally, berberine has exhibited hypolipidemic and hypoglycemic effects and has been used to protect against cardiovascular diseases [8,10]. In comparison, other alkaloids in RC are less studied than berberine. Coptisine has been reported to have anti-tumor, anti-inflammatory, anti-diabetic, and anti-hypercholesterolemic effects by several studies [11,12,13,14]. Palmatine, another classic alkaloid from RC, has been reported to have anti-inflammatory, neuroprotective, and lipid metabolism regulatory effects [15,16,17]. There is a lack of systematic functional studies of the main bioactive alkaloids in RC, and a comparative study of their biological effects has yet to be performed.

The chemical fingerprinting enabled by high-performance liquid chromatography (HPLC) can help evaluate the components and quality of herbs. However, chemical fingerprinting cannot reveal the pharmacological activity of herbs. Therefore, a method that can directly evaluate the biological activities of RC and its main components is needed. As a complement to chemical profiling, biological response fingerprints (BioReFs) have been used to define gene expression patterns for specific drugs [18]. By means of high-throughput technologies, such as microarray and next-generation sequencing, global transcriptional profiles can be generated to reflect the cellular responses to drug treatment.

Our previous study established BioReFs of RC from different growing regions on HepG2 cells [7]. Four types of RC from different regions can be distinguished from Mahoniae Caulis, another herb with similar composition, using BioReFs. In the present study, we aimed to establish the BioReFs of RC crude extract and its representative alkaloids. In addition, we compared their effects on the cell viability of different cell lines and explored their regulatory effects on genes involved in different biological processes. In this way, we analyzed the differences in the typical alkaloids in RC at a systematic level and provided comprehensive information on the pharmaceutical mechanisms of the different alkaloids.

## 2. Results

### 2.1. HPLC Analysis of Alkaloids in Crude RC Extract

**Figure 1:**
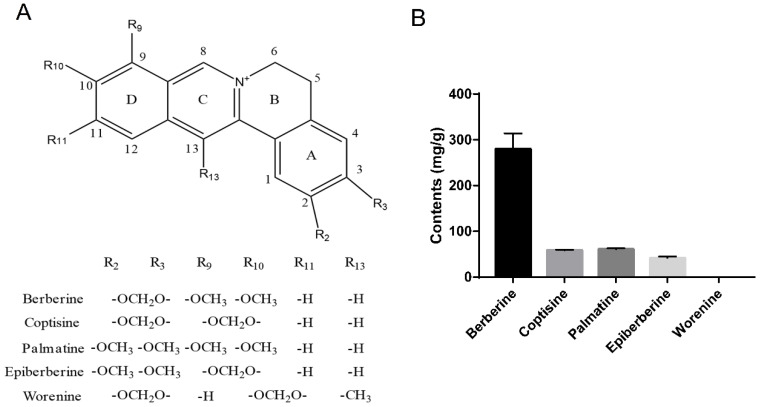
Structures and amount of representative alkaloids, including berberine, coptisine, palmatine, epiberberine, and worenine, in the extracts of Rhizoma Coptidis. (**A**) Structural formulae of alkaloids. (**B**) Contents of representative alkaloids, Data are given as the mean ± SD (*n* = 3).

The contents of five alkaloids in RCE were analyzed using HPLC. The five alkaloids included the four quality markers for RC designated in the China Pharmacopoeia (2015 edition), i.e., berberine, coptisine, palmatine, and epiberberine, in addition to worenine, a relatively rare alkaloid of RC. RCs from three growing regions, i.e., Dayi of Sichuan Province, Lichuan of Hubei Province, and Shizhu of Chongqing City, were included in the analysis. The structural formulae and contents of the five alkaloids are shown in Figure 1. The content of berberine was the highest, accounting for ~28% of the total extract. The following were coptisine and palmatine, each accounting for ~5% and being slightly more abundant than epiberberine. Unfortunately, worenine was not detected in the extracts. Based on the results of HPLC, we focused on three alkaloids, i.e., berberine, coptisine, and palmatine, in the following study considering their relatively high content.

### 2.2. Comparison of the Effects of Alkaloids on the Proliferation of Different Cell Lines

Since several main alkaloids of RC have been reported to have anti-tumor activity, we compared their ability to inhibit cell proliferation in different cell lines. Berberine, coptisine, and palmatine were used to treat the human hepatoma cell line HepG2, the murine macrophage cell line RAW264.7, and the murine fibroblast cell line 3T3-L1 with different concentrations and durations, respectively. The concentrations ranged from 2.5 μM to 100 μM, and the durations included 24 h, 48 h, and 72 h. The cell viability was detected, and the IC_50_ was then calculated.

**Figure 2:**
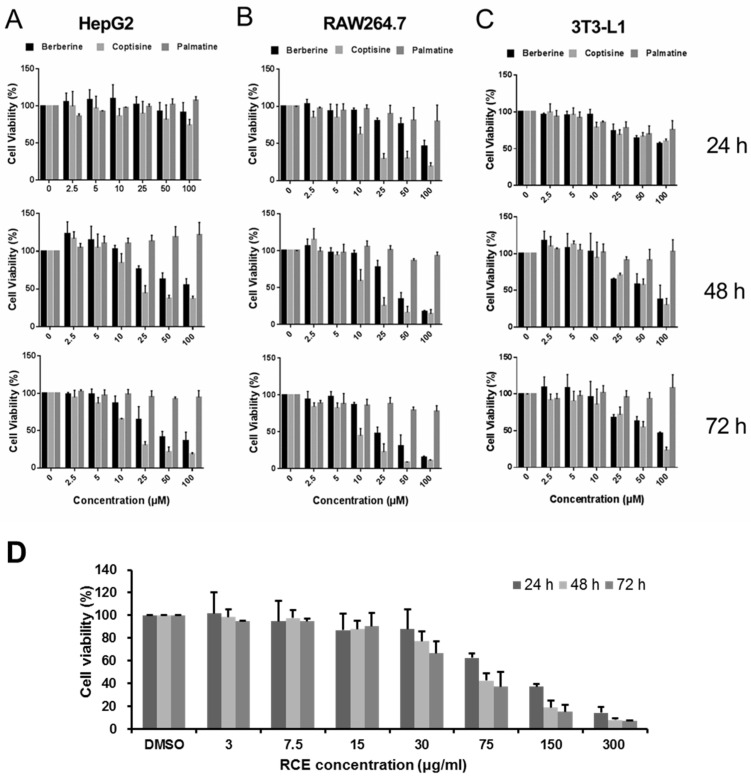
Effects of berberine, coptisine, palmatine, and RCE on cell proliferation in multiple cell lines. Cell viability in response to berberine, coptisine and palmatine treatment at 24 h, 48 h, and 72 h at 2.5 μM, 5 μM, 10 μM, 25 μM, 50 μM, and 100 μM in (**A**) HepG2 cells, (**B**) 3T3-L1 cells, and (**C**) RAW264.7 cells. (**D**) Cell viability of RAW264.7 cells treated with different concentrations of RCE (3 μg/mL,7.5 μg/mL, 15 μg/mL, 30 μg/mL, 75 μg/mL, 150 μg/mL, and 300 μg/mL) at 24, 48, and 72 h. Data are given as the mean ± SD (*n* = 3).

Overall, we found that berberine and coptisine showed cytotoxicity in all three cell lines in a dose- and time-dependent manner (Figure 2). However, within the concentration range used, palmatine showed no inhibitory effect on the cell viability of all three cell lines even at the 72-h time point.

Specifically, in HepG2 cells, we observed that berberine and coptisine hardly impaired cell viability at 24 h (Figure 2A). The IC_50_ of berberine at 48 h and 72 h was 123.42 μM and 47.56 μM, respectively (Table 1). The IC_50_ of coptisine at 48 h and 72 h was 34.88 μM and 18.1 μM, respectively (Table 1). Therefore, coptisine showed a stronger inhibitory effect than berberine in HepG2 cells. In RAW264.7 and 3T3-L1 cells, berberine and coptisine showed inhibitory effects after 24 h, and these effects were even greater at 48 h and 72 h (Figure 2B,C). Similarly, coptisine showed a stronger inhibitory effect than berberine in these two cell lines. In all three cell lines, palmatine showed no cytotoxicity within the concentration and time duration used in this study.

In addition, disparate susceptibility to alkaloids was noted among the three cell lines. Taking the results at 72 h, for example, the IC_50_ values of coptisine in HepG2 cells, RAW264.7 cells, and 3T3-L1 cells were 18.1 μM, 10.29 μM, and 50.63 μM, respectively (Table 1). Therefore, we concluded that RAW264.7 cells were more sensitive to alkaloids than HepG2 and 3T3-L1 cells.

In summary, the three main alkaloids in RC showed different cytotoxicity on the three cell lines we tested. We found that coptisine and berberine had strong inhibitory effects on cell proliferation, while palmatine had no effect on cell proliferation at concentrations as high as 100 μM within 72 h.

### 2.3. Transcriptome Analysis of RCE and Three Representative Alkaloids in RAW264.7 Cells

To further identify the functional differences in the three main alkaloids in RC, RAW264.7 cells were selected for transcriptome analysis due to their high sensitivity to alkaloids. RAW264.7 cells were treated with RCE, berberine, coptisine, and palmatine for 24 h. Because the three main alkaloids have different cytotoxicity, we wanted to use concentrations that did not cause excessive cell death while resulting in adequate disturbance of genome-wide gene expression. Thus, we used 60 μM berberine, 5 μM coptisine, and 80 μM palmitate for microarray analysis, and those concentrations caused approximately 20% cell death, as determined by cell proliferation assay (Figure 2A–C). The cytotoxicity of RCE was also determined by cell viability assay, as shown in Figure 2D. RAW264.7 cells were treated with different concentrations of RCE at 24, 48, and 72 h. We finally used 40 μg/mL RCE for microarray analysis. RNA was collected and subjected to whole-genome transcriptome analysis. The transcriptome dataset is accessible with GEO accession: GSE163032.

**Figure 3:**
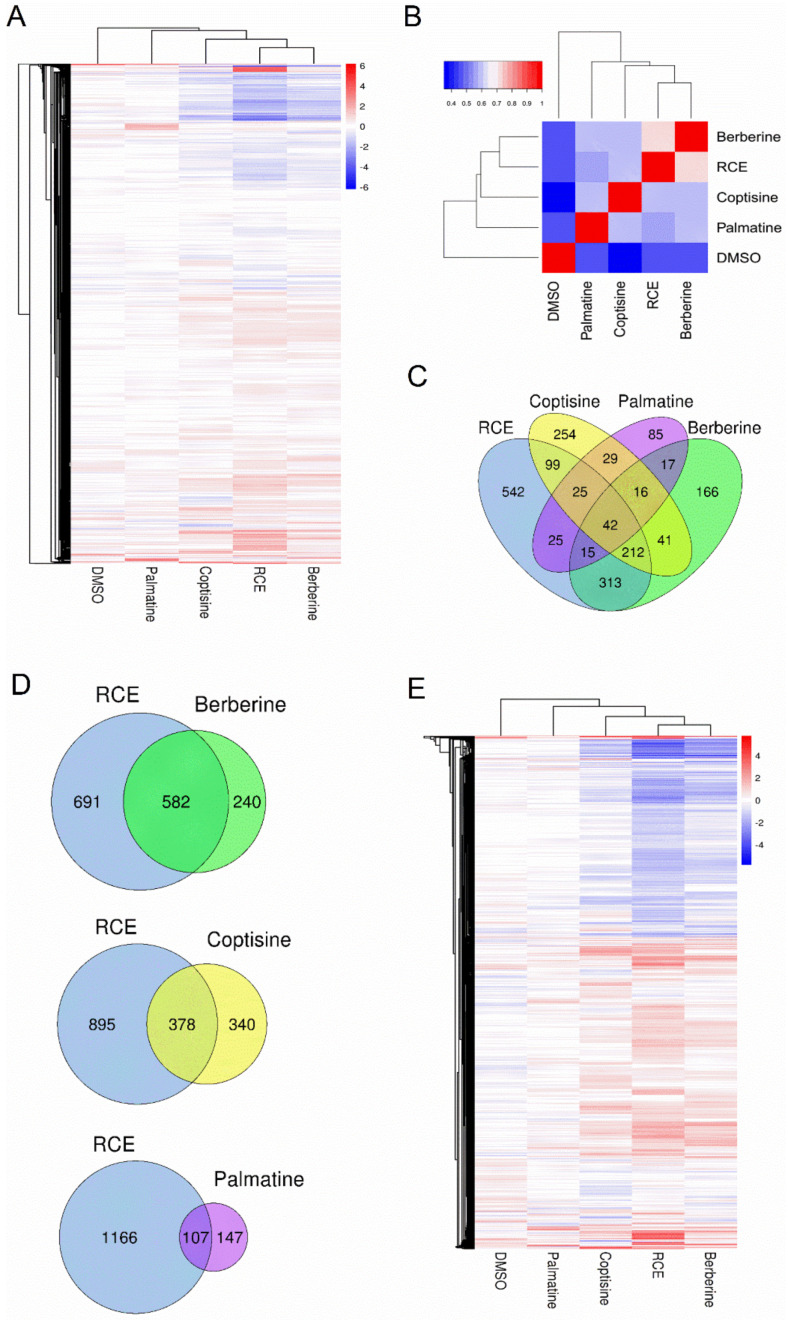
Transcriptome analysis of RCE-, berberine-, coptisine-, and palmatine-treated RAW264.7 macrophages. (**A**) Clustering analysis of all expressed genes in all samples. (**B**) The correlation analysis of gene expression in RCE-, berberine-, coptisine-, palmatine- and DMSO-treated samples. The color indicates the correlation coefficient (blue: low, red: high). (**C**) Venn diagram of differentially expressed genes (DEGs) among RCE-, berberine-, coptisine-, and palmatine-treated RAW264.7 cells. (**D**) Venn diagram of DEGs of RCE vs. berberine, RCR vs. coptisine, and RCE vs. palmatine. (**E**) Hierarchical clustering analysis of DEGs with fold change ≥ 2 in each sample. The color of each spot in the heatmap corresponds to the log 2 transformed fold change vs. the PBS of each gene in four samples, with a gradient of color from blue (low) to red (high).

Clustering analysis of all expressed genes was performed to evaluate the overall similarities and differences in the effects of RCE and three alkaloids on global gene expression of RAW264.7 cells. Among the three alkaloids, the gene expression profile of berberine was the closest to that of RCE, suggesting that berberine could better represent the effect of RCE on gene expression than the other two alkaloids (Figure 3A). The clustering pattern also showed that palmatine clustered far from RCE and the other alkaloids, very close to DMSO, suggesting that palmatine had a mild effect on gene expression in RAW264.7 cells. Correlation analysis also suggested the same conclusion—that berberine was most similar to RCE, while palmatine was the least similar one (Figure 3B).

Using two-fold as a cut-off value, there were 1273 DEGs between the RCE group and the DMSO group. Among them, 585 were upregulated, and 691 were downregulated (Table 2). There were 822, 718, and 254 DEGs of berberine, coptisine, and palmatine, respectively. Comparing all the DEGs, we found that berberine presented the highest degree of overlap with RCE, with an overlapping ratio of 70.8% (582/822), which was consistent with the clustering results (Figure 3C,D). The overlapping ratio for DEGs of coptisine vs. RCE was 52.6%, and that for palmatine vs. RCE was 42.1% (Figure 3D). Clustering analysis of the combined DEGs from each treatment showed the same pattern of all expressed genes: berberine was most similar to RCE, while palmatine was the least similar one (Figure 3E).

### 2.4. Pathway Analysis of RCE and Three Representative Alkaloids in RAW264.7 Cells

**Figure 4:**
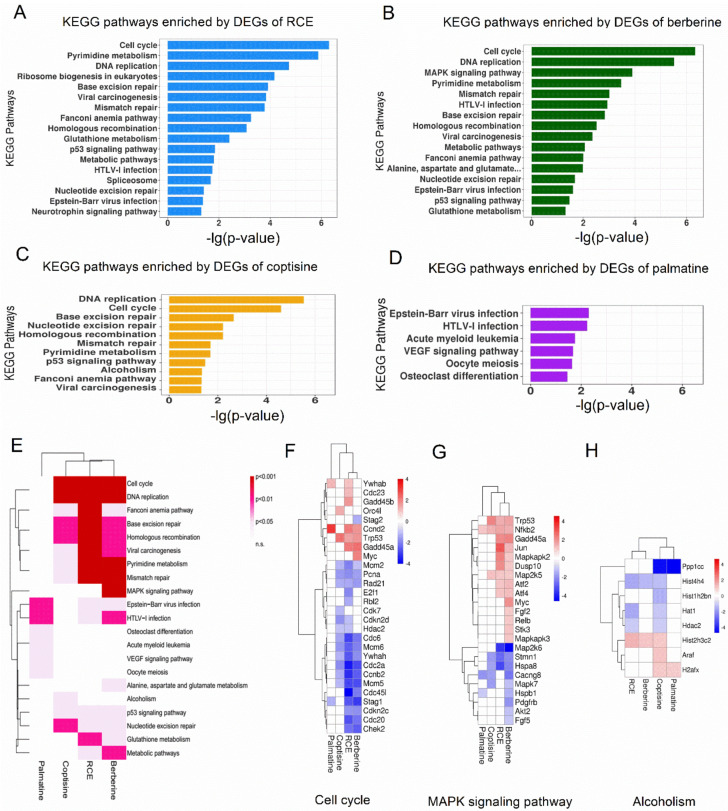
Pathway enrichment analysis of RCE-, berberine-, coptisine-, and palmatine-treated RAW264.7 macrophages. (**A**) Significantly enriched KEGG pathways for DEGs of RCE, (**B**) berberine, (**C**) coptisine, and (**D**) palmatine (*p* < 0.05). (**E**) Clustering analysis of the KEGG pathways enriched by DEGs of RCE, berberine, coptisine, and palmatine. The color indicates the *p*-value. The redder the color, the smaller the *p*-value. Heatmap of differential expression of genes implicated in (**F**) the cell cycle, (**G**) MAPK signaling pathway, and (**H**) alcoholism. The color of each spot in the heatmap corresponds to the log 2 transformed fold change vs. DMSO of each gene in four samples, with a gradient of color from blue (low) to red (high).

We performed KEGG pathway enrichment analysis using DEGs of RCE, berberine, coptisine, and palmatine. The top hits for RCE, berberine, and coptisine included cell cycle, DNA replication, base excision repair, homologous recombination, etc. (Figure 4A–C). However, the number of enriched pathways of palmatine was small, and the top hits for palmatine were EB virus infection and HTLV1 infection (Figure 4D). Clustering analysis of the enriched pathways of DEGs of RCE, berberine, coptisine, and palmatine indicated that berberine and coptisine were similar to RCE to some degree, while palmatine had its own pattern (Figure 4E). Thus far, we concluded that berberine and coptisine, as the main bioactive components of RCE and palmatine, showed low bioactivity in RAW264.7 cells.

Among the enriched pathways, we found some pathways commonly affected by RCE, berberine, and coptisine, such as the cell cycle, as shown in Figure 4F. This result was consistent with the cell proliferation assay showing that coptisine and berberine both inhibited proliferation in RAW264.7 cells. We also noticed that some pathways were only enriched by berberine, such as the MAPK signaling pathway (Figure 4G). We can see that a number of genes involved in the MAPK signaling pathway were orchestrated by berberine, while coptisine only regulated some of them. Moreover, there existed pathways uniquely enriched by coptisine, such as alcoholism (Figure 4H). This suggested that berberine and coptisine shared some common activities but also had their own specific functions.

### 2.5. Comparative Analysis of Berberine and Coptisine on Genes Involved in Different Biological Processes

**Figure 5:**
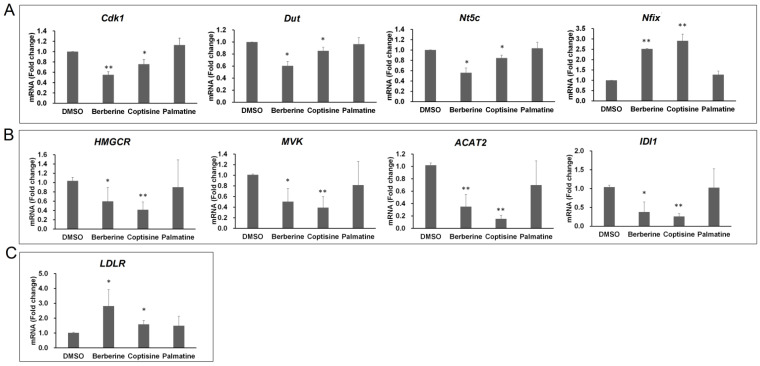
qRT-PCR analysis of genes involved in different biological processes. (**A**) qRT-PCR analysis of genes related to the cell cycle in RAW264.7 cells after exposure to berberine, coptisine, and palmatine for 24 h. (**B**) qRT-PCR analysis of genes related to the MVA pathway in HepG2 cells after exposure to berberine, coptisine, and palmatine for 24 h. (**C**) qRT-PCR analysis of *LDLR* in HepG2 cells after exposure to berberine, coptisine, and palmatine for 24 h. Data are given as the mean ± SD (*n* = 3). * *p* < 0.05, ** *p* < 0.01 vs. DMSO group.

Based on the proliferation assay and the transcriptome analysis, we concluded that berberine and coptisine both had cytotoxic effects. We selected several representative genes related to the cell cycle and checked their expression changes in different groups. Cyclin-dependent kinase 1 (*Cdk1*), deoxyuridine triphosphatase (*Dut*), 5′,3′-nucleotidase, cytosolic (*Nt5c*), and nuclear factor I/X (*Nfix*) were selected for qRT-PCR validation. CDK1 is essential for G1/S and G2/M phase transitions of the eukaryotic cell cycle [19]. DUT and NT5C are enzymes involved in nucleotide metabolism and are associated with the prognosis of cancer patients [20,21]. NFIX is part of a family of highly conserved DNA-binding proteins that function as transcriptional activators and/or repressors, playing a significant role in hematopoiesis, muscle development, and brain development [22]. Not surprisingly, the expression levels of *Cdk1*, *Dut*, *Nt5c* were all downregulated by berberine and coptisine, and berberine decreased the genes by about two-fold while the reduction effect of coptisine was marginal. *Nfix* was upregulated by both berberine and coptisine at 60 μM in RAW264.7 cells (Figure 5A). Palmatine, included as a control, did not affect their expression at the same concentration.

As revealed by the microarray on RAW264.7 cells, RCE interfered with metabolic pathways. To compare the effect of berberine and coptisine on metabolism, we switched to a better cellular model, the human hepatoma cell line HepG2. In our previous study, we demonstrated that RCE regulated the mevalonate (MVA) pathway, the first portion of the de novo cholesterol synthesis pathway, and resulted in reduced cholesterol synthesis in HepG2 cells [7]. We also showed that both berberine and coptisine downregulated diphosphomevalonate decarboxylase (*MVD*) and hydroxymethylglutaryl-CoA synthase (*HMGCS1*) expression in a dose-dependent manner [7].

In the current study, we extended the gene panel and checked the expression levels of 3-hydroxy-3-methylglutaryl-coenzyme A reductase (*HMGCR*), mevalonate kinase (*MVK*), *acetyl-CoA acetyltransferase (ACAT2*), and isopentenyl-diphosphate delta-isomerase 1 (*IDI1*) following exposure to 40 μM berberine, coptisine, or palmatine in HepG2 cells. Similarly, we found that berberine and coptisine decreased the expression of all four genes, while palmatine did not (Figure 5B). The inhibitory effect was even stronger for coptisine than for berberine.

We tested another gene, low-density lipoprotein receptor (*LDLR*), in response to berberine, coptisine, and palmatine treatment. LDLR is another critical gene for cholesterol homeostasis, and it mediates LDL-c clearance in blood. We found that berberine increased *LDLR* mRNA levels by 2.81-fold, while coptisine only resulted in a marginal increase of 1.58-fold (Figure 5C). Palmatine increased the expression of *LDLR,* but the change was not statistically significant at 40 μM.

In summary, we concluded that berberine and coptisine were both key regulators of MVA pathway genes and that the effects of coptisine were stronger than those of berberine. However, berberine showed a stronger effect in upregulating *LDLR* than did coptisine.

## 3. Discussion

The aim of our study was to analyze the differences in the main alkaloids of RC from different aspects. We began with a chemical content analysis based on HPLC. We tested five alkaloids and found that the content of berberine was the highest, followed by coptisine and palmatine, and then epiberberine. This overall trend was consistent with studies by other groups [23,24]. Worenine was not detected in our extract, likely due to its low initial content in RC. The content of worenine in crude RCE was detected as only 0.02~0.03 mg/g [24], and the actual yield might have been affected by the extraction process.

Different studies have confirmed the cytotoxicity of RC and some of its alkaloids. Yi et al. tested the cytotoxicity of berberine, coptisine, palmatine, and epiberberine on HepG2 and 3T3-L1 cells [25]. They found that the toxicity of berberine was the highest and that of palmatine was the smallest, based on a cell assay at 24 h [25]. However, our data indicated that coptisine had a stronger inhibitory effect on the proliferation of HepG2, 3T3-L1, and RAW264.7 cells. These differences were likely caused by the different cell sources and cell statuses. In addition, we tested cell proliferation at multiple time points. In our experiments, the effects of berberine and coptisine on HepG2 and 3T3-L1 cells were comparable at 24 h of treatment at each concentration; however, coptisine began to exert stronger cytotoxicity when the treatment time was extended to 48 and 72 h. Overall, our data support the anti-proliferation activity of berberine and coptisine in vitro, but this activity was not observed for palmatine.

In addition, we established the BioReFs of RCE and three representative alkaloids in RAW264.7 cells. The transcriptome analysis helped us to compare RCE and three alkaloids from a systematic level. We concluded that berberine was most similar to RCE, followed by coptisine, while palmatine was the least similar one. This conclusion is not surprising, but the clustering results help us to directly visualize the similarity between RCE and its alkaloids. Based on pathway analysis, it is not surprising that RCE, berberine, and coptisine affected common pathways, such as the cell cycle and DNA replication. However, palmatine showed a mild effect on gene expression. This was supported by the cell proliferation assay and qRT-PCR analysis of cell cycle-related genes.

We also noticed that berberine and coptisine had their own specific effects on genes and pathways. Considering our previous studies on the regulatory effect of RCE on lipid metabolism, we explored the roles of berberine, coptisine, and palmatine on genes involved in cholesterol biosynthesis and LDL-c uptake, i.e., LDLR. We found that coptisine was very efficient in inhibiting MVA genes. However, berberine was a strong inducer of LDLR expression.

Our previous work revealed that RCE could downregulate a cluster of genes in the MVA pathway [7]. However, this effect was not mediated by suppressing the coincident regulator sterol regulatory element-binding protein 2 (SREBP2) or by promoting the phosphorylation of extracellular signal-regulated kinase (ERK) [7]. Here, we showed that coptisine is even stronger than the other compounds in regulating MVA pathways, although the underlying mechanism is not clear. This suggests that alkaloids besides berberine also have important pharmaceutical activity in certain aspects, although berberine is regarded as the most important active constituent of RC.

Different studies have reported that berberine could regulate LDLR in multiple ways, and we showed that berberine strongly upregulated LDLR, with much greater effects than those of coptisine. Because berberine and coptisine have superiority in different pathways of cholesterol metabolism, treatment with the combination of berberine and coptisine may be a good choice to lower cholesterol. Kou et al. suggested that the combination of berberine, coptisine, palmatine, epiberberine, and jatrorrhizine showed synergetic cholesterol-lowering effects in HepG2 cells and hypercholesterolemic hamsters [23]. In their study, they showed that the combination of five main alkaloids resulted in stronger LDLR upregulation and HMGCR downregulation than each single alkaloid. It is worth further studying the combination of berberine and coptisine for their lipid-lowering effects in vivo and exploring the underlying mechanisms.

Coptisine, berberine, and palmatine were quaternary protoberberine alkaloid with different substituents at C-2, C-3 on ring A, and C-9, C-10 on ring D (Figure 1A). According to our research, the effects on anti-tumor and cholesterol regulation of compounds with methylenedioxy group at C-2, C-3 on ring A (coptisine, berberine) were significantly stronger than those with di-methoxy group at C-2, C-3 on ring A (palmatine). Methylenedioxy or di-methoxy group at C-9, C-10 on ring D may lay the foundation of the difference between berberine and coptisine in lipid regulation.

In summary, we analyzed the differences in the main alkaloids of RC from different aspects. Especially, we introduced the BioReFs to compare the bioactivity of different RC alkaloids. We found that berberine is the most important active constituent and could partially represent the bioactivity of RC extract; meanwhile, berberine and coptisine are the primary cytotoxic component of RC. Berberine and coptisine had different regulatory effects on genes involved in lipid metabolism, probably due to their different structure, which provided some clues for lead optimization.

## 4. Materials and Methods

### 4.1. Cells and Agents

The human hepatoma cell line HepG2, murine macrophage cell line RAW264.7, and murine fibroblast cell line 3T3-L1 were obtained from the National Infrastructure of Cell Line Resource (Beijing, China). All cell lines were cultured in high glucose DMEM medium containing 10% fetal bovine serum and 1% antibiotic mixture of penicillin (100 U/mL) and streptomycin (100 mg/mL).

Rhizomes of *Coptis chinensis* Franch from 3 regions (Dayi of Sichuan Province, Lichuan of Hubei Province and Shizhu of Chongqing City, China) were purchased from the Anguo city traditional Chinese medicine market. The main alkaloid standards (berberine, coptisine, palmatine, epiberberine, and worenine) were purchased from the National Institutes for Food and Drug Control, Beijing, China.

### 4.2. Preparation of Crude Rhizoma Coptidis Extract (RCE)

All RC samples were balanced and ground into powder. The crude powder was backflow extracted with 70% ethanol twice at a 1:10 (*w*/*v*) proportion for one hour each time. The resulting extract was concentrated under reduced pressure and freeze-dried into powder. The extracted powder was accurately weighed and redissolved in DMSO as a stock solution.

### 4.3. HPLC Analysis

The HPLC analysis was performed as previously described [7]. Briefly, the RCE was dissolved in 50% methanol, and the components were detected using an Agilent 1260 Series HPLC system (Agilent Technologies, Santa Clara, CA, USA). The system consisted of a quaternary pump system, a thermostatic column compartment, an autosampler, and a photodiode array detector. ChemStation (Rev.B.02.01) software was used for the control of the HPLC system, data acquisition, and processing. An Agilent Zorbax SB-C18 (250 mm × 4.6 mm i.d., 5 µm) column was used for chromatographic separation. The mobile phase (flow rate of 1.0 mL/min) was composed of (A) acetonitrile and (B) water containing 0.25% SDS (*w*/*v*), 0.25% KH_2_PO_4_ (*w*/*v*), and 0.1% phosphoric acid (*v*/*v*). The program was an isocratic elution at 44% of solution A for 20 min with an injection volume of 10 µL, and the detection wavelength was 345 nm. A mixed standard solution of five reference standards (berberine, coptisine, palmatine, epiberberine, and worenine) was used to obtain the standard curves. The content of each alkaloid in RCE was calculated based on the standard curves. RCs from three growing regions (Dayi of Sichuan Province, Lichuan of Hubei Province and Shizhu of Chongqing City, China) were used to obtain the average content.

### 4.4. Cell Proliferation Assay

Cells were seeded in 96-well plates and treated with drugs at different concentrations. After incubation, the media were removed, and CellTiter^®^ 96 AQ_ueous_ One Solution Reagent (Promega, Madison, WI, USA) was added and incubated at 37 °C for 3 h. Absorbance at 490 nm was measured using a Spectromax spectrophotometer. The cell viability rate was calculated as follows: (OD_treatment_ − OD_blank_)/(OD_control_ − OD_blank_)] × 100%. All experiments were performed in triplicate and repeated three times independently.

### 4.5. RNA Extraction, cDNA Synthesis, and Quantitative Real-Time PCR (qRT-PCR)

Total RNA was prepared using TRIzol reagent (Invitrogen, Carlsbad, CA, USA) according to the manufacturer’s instructions. Total RNA (1 μg) was treated with a Turbo DNase free kit (Thermo Fisher Scientific, Waltham, MA, USA) and reverse transcribed using a High-Capacity RNA-to-cDNA Kit (Thermo Fisher Scientific Baltics UAB, Vilnius, Lithuania). qRT-PCR reactions were carried out in duplicate with Fast SYBR^®^ Green Master Mix (Kapa Biosystems, Wilmington, MA, USA). The thermal cycling conditions of PCR were as follows: 95 °C for 3 min, 95 °C for 10 s, 60 °C for 30 s, and 40 cycles of amplification. The relative expression was calculated using the following formula: Fold change = 2^−ΔΔCT^, where ΔΔCT = ΔCT_sample_ − ΔCT_control_; ΔCT = average CT _test gene_ − average CT _GAPDH_, and CT stands for cycle threshold. All primer sequences are available upon request.

### 4.6. Gene Expression Profiling and Analysis

Total RNA was used to produce Cy5-dCTP- or Cy3-dCTP-labelled cDNA using Eberwine’s linear RNA amplification method, according to a previously published protocol [26]. The CapitalBio 36K mouse genome oligo array was used for hybridization (CapitalBio Corporation, Beijing, China). The dual-channel platform included hybridization of the samples treated with RCE, berberine, coptisine, and plamatine and those treated with PBS. Microarrays were then scanned using a LuxScan^TM^ Scanner (CapitalBio, Beijing, China). A corrected *p*-value of 0.05 and a |log_2_ (fold change)| of 1 were set as the threshold for statistically significant differential expression.

To identify the functional pathways associated with differentially expressed genes (DEGs), online analytical tools such as the Database for Annotation, Visualization, and Integrated Discovery (DAVID, http://david.abcc.ncifcrf.gov/, accessed on 1 September 2019) were used.

### 4.7. Statistics

Statistical analysis of experimental data was conducted using Student’s *t*-test. Values were expressed as the mean ± standard deviation (SD). A *p*-value < 0.05 was accepted as statistically significant.

## 5. Conclusions

The current study analyzed the differences in the typical alkaloids of RC at a systematic level. BioReFs have been established for RCE, berberine, coptsine, and palmatine in RAW264.7 cells. Berberine was identified as most similar of the alkaloids to RCE and could partially represent the bioactivity of RCE. Both the transcriptome analysis and cell proliferation assay suggested that berberine and coptisine exhibit cytotoxicity. In addition, we found that berberine and coptisine had different regulatory effects on genes involved in lipid metabolism. This study provides comprehensive information on the pharmaceutical mechanisms of the different RC alkaloids and proposes combinatory uses of them for the treatment of diseases.

## Figures and Tables

**Table 1:** The IC_50_ (μM) of berberine, coptisine, and palmate in different cell lines at different time points.

Cell Line	HepG2			RAW264.7			3T3-L1		
Time (h)	24	48	72	24	48	72	24	48	72
Berberine	N/A	123.4	47.56	104.8	41.93	28.27	120.8	76.45	77.97
Coptisine	454.7	34.88	18.10	16.82	15.38	10.29	152.9	53.26	50.63
Palmatine	N/A	N/A	24.33 × 10^4^	883.4	7292	29.16 × 10^3^	1043	N/A	N/A

**Table 2:** Summary of the DEG numbers in cells treated with RCE, berberine, coptisine, and palmatine.

Treatment	Total DEGs Number	Upregulated Gene Number	Downregulated Gene Number
RCE	1273	585	691
Berberine	822	346	478
Coptisine	718	359	360
Palmatine	254	124	131

## Data Availability

Available on request.

## Sample Availability

Samples of the compounds are available from the corresponding author.
